# (η^6^-Benzene)­dichlorido(dicyclo­hexyl­phenyl­phosphane)ruthenium(II) benzene sesquisolvate

**DOI:** 10.1107/S1600536812044674

**Published:** 2012-11-03

**Authors:** Alfred Muller, Wade L. Davis

**Affiliations:** a Research Centre for Synthesis and Catalysis, Department of Chemistry, University of Johannesburg (APK Campus), PO Box 524, Auckland Park, Johannesburg, 2006, South Africa

## Abstract

The asymmetric unit of the title compound, [RuCl_2_(C_6_H_6_)(C_18_H_27_P)]·1.5C_6_H_6_, contains one mol­ecule of the Ru^II^ complex and one and a half solvent molecules as one of these is located about a centre of inversion. The Ru^II^ atom has a classical three-legged piano-stool environment being coordinated by an η^6^-benzene ligand [Ru—centroid = 1.6964 (6) Å], two chloride ligands with an average Ru—Cl bond length of 2.4138 (3) Å and a dicyclo­hexyl­phenyl­phosphane ligand [Ru—P = 2.3786 (3) Å]. The effective cone angle for the phosphane was calculated to be 158°. In the crystal, weak C—H⋯Cl hydrogen bonds link the Ru^II^ complexes into centrosymmetric dimers. The crystal packing exhibits intra- and inter­molecular C—H⋯π inter­actions resulting in a zigzag pattern in the [101] direction.

## Related literature

For background to the catalytic activity of Ru^II^–arene complexes, see: Chen *et al.* (2002 ▶); Crochet *et al.* (2003 ▶); Aydemir *et al.* (2011 ▶); Wang *et al.* (2011 ▶). For ring-opening metathesis polymerization with Ru–arene complexes, see: Stumpf *et al.* (1995 ▶). For background to cone angles, see: Tolman (1977 ▶); Otto (2001 ▶). For a description of the Cambridge Structural Database, see: Allen (2002 ▶).
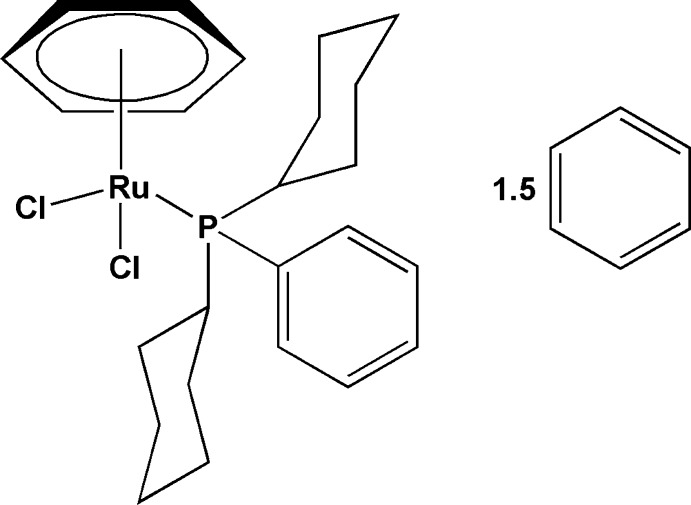


## Experimental

### 

#### Crystal data

[RuCl_2_(C_6_H_6_)(C_18_H_27_P)]·1.5C_6_H_6_
*M* *_r_* = 641.61
Triclinic, 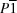

*a* = 10.0893 (8) Å
*b* = 10.8325 (9) Å
*c* = 14.4937 (12) Å
α = 90.346 (2)°
β = 91.748 (1)°
γ = 106.979 (1)°
*V* = 1514.1 (2) Å^3^
*Z* = 2
Mo *K*α radiation
μ = 0.77 mm^−1^
*T* = 100 K
0.43 × 0.17 × 0.16 mm

#### Data collection

Bruker APEX DUO 4K CCD diffractometer
Absorption correction: multi-scan (*SADABS*; Bruker, 2008 ▶) *T*_min_ = 0.734, *T*_max_ = 0.887
49345 measured reflections
7589 independent reflections
7093 reflections with *I* > 2σ(*I*)
*R*_int_ = 0.022

#### Refinement

*R*[*F* ^2^ > 2σ(*F* ^2^)] = 0.018
*wR*(*F* ^2^) = 0.048
*S* = 1.03
7589 reflections
334 parameters
H-atom parameters constrained
Δρ_max_ = 0.54 e Å^−3^
Δρ_min_ = −0.43 e Å^−3^

### 

Data collection: *APEX2* (Bruker, 2011 ▶); cell refinement: *SAINT* (Bruker, 2008 ▶); data reduction: *SAINT* and *XPREP* (Bruker, 2008 ▶); program(s) used to solve structure: *SIR97* (Altomare *et al.*, 1999 ▶); program(s) used to refine structure: *SHELXL97* (Sheldrick, 2008 ▶); molecular graphics: *DIAMOND* (Brandenburg & Putz, 2005 ▶); software used to prepare material for publication: *publCIF* (Westrip, 2010 ▶) and *WinGX* (Farrugia, 1999 ▶).

## Supplementary Material

Crystal structure: contains datablock(s) global, I. DOI: 10.1107/S1600536812044674/cv5349sup1.cif

Structure factors: contains datablock(s) I. DOI: 10.1107/S1600536812044674/cv5349Isup2.hkl

Additional supplementary materials:  crystallographic information; 3D view; checkCIF report

## Figures and Tables

**Table 1:** Hydrogen-bond geometry (Å, °) *Cg*1 and *Cg*2 are the centroids of the C19–C24 and C31–C33/C31′–C33′) benzene rings.

*D*—H⋯*A*	*D*—H	H⋯*A*	*D*⋯*A*	*D*—H⋯*A*
C3—H3⋯Cl2^i^	0.95	2.76	3.6307 (13)	153
C4—H4⋯Cl1^i^	0.95	2.7	3.6209 (13)	163
C6—H6⋯*Cg*1	0.95	2.78	3.5086 (14)	135
C2—H2⋯*Cg*2^ii^	0.95	2.73	3.5869 (15)	150

Symmetry codes: (i) 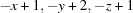; (ii) 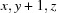.

**Fig. 1.**
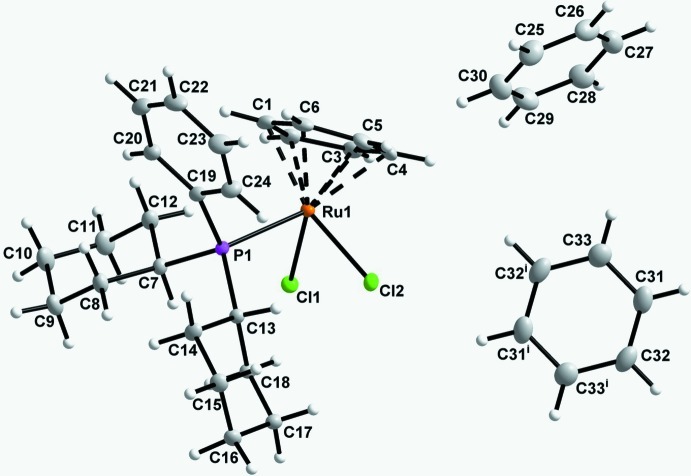
A view of the title complex, showing the atom-numbering scheme and 50% probability displacement ellipsoids. Accented lettering indicate atoms generated by symmetry code: 1 - x,1 - y,1 - z.

**Fig. 2.**
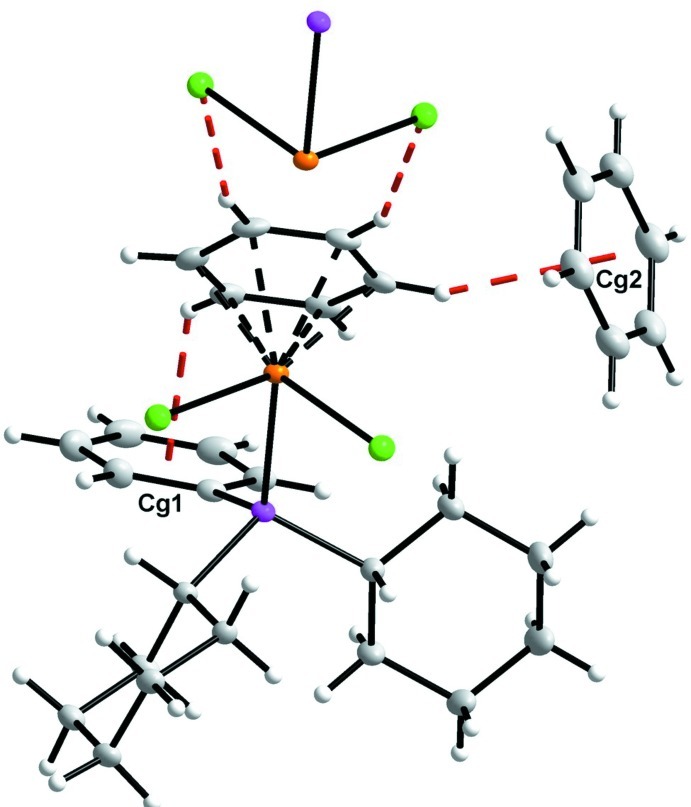
Packing diagram showing the C—H···Cl/π interactions (indicated by red dashed lines).

## supplementary crystallographic information

### Comment

The activity of the half-sandwich Ru(II)-arene complexes are well known in the catalytic transfer hydrogenation of carbonyl compounds (Chen *et al.*, 2002; Crochet *et al.*, 2003; Aydemir *et al.*, 2011; Wang *et al.*, 2011) and for ring-opening metathesis polymerization (Stumpf *et al.*, 1995). Reported here is the η^6^-Ru compound containing the phosphane, PCy_2_Ph, where Cy = C_6_H_11_ and Ph = C_6_H_5_ as part of our ongoing structural investigation into these type of complexes.

The title compound crystallizes in the triclinic space group *P*1 (*Z*=2), with its molecules adopting a classical three-legged piano-stool environment observed for these type of complexes. Each Ru complex co-crystallizes with sesqui benzene solvate molecules due to one of the solvate being situated on an inversion centre (see Fig. 1). The coordination sphere of the ruthenium is occupied by a benzene, dicyclohexylphenylphosphane and two chloride atoms. The distance between Ru and the centroid of the π-bonded η^6^-benzene ligand is 1.6964 (6) Å and the mean Ru—C bond distance is 2.2099 (13) Å. The coordination of the remaining ligands to the Ru atom shows a slight deviation from the typical octahedral geometry with Cl—Ru—Cl = 88.07 (11) and Cl—Ru—P = 87.12 (11), 90.97 (2)°. The bond distances of Ru—P = 2.3786 (3) and Ru—Cl(avg.) = 2.4138 (3) Å are within normal ranges (Allen, 2002).

The steric demand of phosphane ligands is usually described with the use of the Tolman cone angle model (Tolman, 1977). In the present study we make use of an adaptation of this model whereby the geometry obtained from the title compound (and adjusting the Ru—P bond distance to 2.28 Å) is used to calculate an effective cone angle (Otto, 2001). The value obtained with this method is 158°, which is marginally smaller that the average effective cone angle value calculated from literature observations of the phosphane ligand. Data extracted from the Cambridge Structural Database (Allen, 2002) shows an average cone angle of 165° for the phosphane from 31 hits, containing 45 useable observations with a standard deviation of ±6° and a spread from 148° to 180°.

The slightly smaller cone angle value obtained for the phosphane ligand in the title compound could be due to a crowded metal coordination environment as well as several C–H···Cl and C–H···π interactions that are observed (see Fig. 2, Table 1 for a graphical representation of the interactions).

### Experimental

[(C_6_H_6_)RuCl_2_]_2_ (50.0 mg, 0.10 mmol) and dicyclohexylphenylphosphane (60.2 mg, 0.22 mmol) in benzene (25 ml) were refluxed under argon for 4 h. The resulting red solution was cooled and filtered to obtain the title complex as orange needles suitable for a single-crystal X-ray study. Analytical data: ^31^P {H} NMR (CDCl_3_, 161.99 MHz): δ (p.p.m.) 24.74 (s, 1P). ^1^H NMR (CDCl_3_, 400 MHz): δ (p.p.m.) 1.23 - 2.45 (m, 22H, 2×C_6_H_11_); 5.27 (s, 6H, C_6_H_6_); 7.43 (m, 3H, Ar—H of C_6_H_5_); 7.77 (t, 2H, Ar—H of C_6_H_5_); 7.34 (s, 6H, Ar—H of C_6_H_6_ co-crystallized solvate)

### Refinement

The aromatic, methine and methylene H atoms were placed in geometrically idealized positions (C—H = 0.95–1.00) and allowed to ride on their parent atoms, with *U*_iso_(H) = 1.2*U*_eq_(C).

### Crystal data

**Table:**

[RuCl_2_(C_6_H_6_)(C_18_H_27_P)]·1.5C_6_H_6_	*Z* = 2
*M**_r_* = 641.61	*F*(000) = 666
Triclinic, *P*1	*D*_x_ = 1.407 Mg m^−^^3^
Hall symbol: -P 1	Mo *K*α radiation, λ = 0.71073 Å
*a* = 10.0893 (8) Å	Cell parameters from 9969 reflections
*b* = 10.8325 (9) Å	θ = 2.4–28.4°
*c* = 14.4937 (12) Å	µ = 0.77 mm^−^^1^
α = 90.346 (2)°	*T* = 100 K
β = 91.748 (1)°	Needle, orange
γ = 106.979 (1)°	0.43 × 0.17 × 0.16 mm
*V* = 1514.1 (2) Å^3^	

### Data collection

**Table:**

Bruker APEX DUO 4K CCD diffractometer	7589 independent reflections
Radiation source: sealed tube	7093 reflections with *I* > 2σ(*I*)
Graphite monochromator	*R*_int_ = 0.022
Detector resolution: 8.4 pixels mm^-1^	θ_max_ = 28.4°, θ_min_ = 2.0°
φ and ω scans	*h* = −12→13
Absorption correction: multi-scan (*SADABS*; Bruker, 2008)	*k* = −14→14
*T*_min_ = 0.734, *T*_max_ = 0.887	*l* = −19→19
49345 measured reflections	

### Refinement

**Table:**

Refinement on *F*^2^	Primary atom site location: structure-invariant direct methods
Least-squares matrix: full	Secondary atom site location: difference Fourier map
*R*[*F*^2^ > 2σ(*F*^2^)] = 0.018	Hydrogen site location: inferred from neighbouring sites
*wR*(*F*^2^) = 0.048	H-atom parameters constrained
*S* = 1.03	*w* = 1/[σ^2^(*F*_o_^2^) + (0.0236*P*)^2^ + 0.8124*P*] where *P* = (*F*_o_^2^ + 2*F*_c_^2^)/3
7589 reflections	(Δ/σ)_max_ = 0.006
334 parameters	Δρ_max_ = 0.54 e Å^−^^3^
0 restraints	Δρ_min_ = −0.43 e Å^−^^3^

### Special details

**Table:**

Experimental. The intensity data was collected on a Bruker Apex DUO 4 K CCD diffractometer using an exposure time of 10 s/frame. A total of 3975 frames were collected with a frame width of 0.5° covering up to θ = 28.39° with 99.8% completeness accomplished.
Geometry. All e.s.d.'s (except the e.s.d. in the dihedral angle between two l.s. planes) are estimated using the full covariance matrix. The cell e.s.d.'s are taken into account individually in the estimation of e.s.d.'s in distances, angles and torsion angles; correlations between e.s.d.'s in cell parameters are only used when they are defined by crystal symmetry. An approximate (isotropic) treatment of cell e.s.d.'s is used for estimating e.s.d.'s involving l.s. planes.
Refinement. Refinement of *F*^2^ against ALL reflections. The weighted *R*-factor *wR* and goodness of fit *S* are based on *F*^2^, conventional *R*-factors *R* are based on *F*, with *F* set to zero for negative *F*^2^. The threshold expression of *F*^2^ > σ(*F*^2^) is used only for calculating *R*-factors(gt) *etc*. and is not relevant to the choice of reflections for refinement. *R*-factors based on *F*^2^ are statistically about twice as large as those based on *F*, and *R*- factors based on ALL data will be even larger.

### Fractional atomic coordinates and isotropic or equivalent isotropic displacement parameters (Å^2^)

**Table:**

	*x*	*y*	*z*	*U*_iso_*/*U*_eq_	
C13	0.23801 (12)	0.90129 (11)	0.13276 (8)	0.0128 (2)	
H13	0.2204	0.8214	0.1704	0.015*	
Ru1	0.291263 (9)	1.021309 (9)	0.364239 (6)	0.01185 (3)	
Cl2	0.35692 (3)	0.82818 (3)	0.330250 (19)	0.01576 (6)	
Cl1	0.51362 (3)	1.14613 (3)	0.310072 (19)	0.01539 (6)	
P1	0.20357 (3)	1.02428 (3)	0.210321 (19)	0.01140 (6)	
C21	−0.20563 (14)	1.02643 (15)	0.22475 (9)	0.0240 (3)	
H21	−0.2563	1.0878	0.2254	0.029*	
C24	−0.05677 (13)	0.84778 (13)	0.22298 (9)	0.0199 (2)	
H24	−0.0066	0.786	0.2228	0.024*	
C7	0.27287 (12)	1.18293 (11)	0.15452 (8)	0.0141 (2)	
H7	0.3714	1.1894	0.1416	0.017*	
C3	0.34809 (14)	1.10795 (13)	0.50804 (8)	0.0205 (2)	
H3	0.4342	1.1512	0.5383	0.025*	
C19	0.01393 (12)	0.97891 (12)	0.21045 (8)	0.0151 (2)	
C12	0.28055 (14)	1.29985 (12)	0.21791 (8)	0.0189 (2)	
H12A	0.1856	1.3019	0.2314	0.023*	
H12B	0.3285	1.2914	0.2771	0.023*	
C15	0.17462 (13)	0.74268 (12)	−0.00063 (8)	0.0184 (2)	
H15A	0.1478	0.6683	0.0413	0.022*	
H15B	0.1177	0.7191	−0.0585	0.022*	
C6	0.08641 (13)	0.98146 (14)	0.42244 (8)	0.0207 (3)	
H6	−0.0029	0.9389	0.3963	0.025*	
C5	0.16284 (14)	0.91076 (14)	0.47028 (9)	0.0217 (3)	
H5	0.1282	0.8193	0.472	0.026*	
C9	0.28183 (16)	1.32479 (12)	0.01475 (9)	0.0227 (3)	
H9A	0.3764	1.3232	−0.0004	0.027*	
H9B	0.2323	1.334	−0.0436	0.027*	
C23	−0.19916 (14)	0.80717 (14)	0.23563 (10)	0.0247 (3)	
H23	−0.2455	0.7181	0.2432	0.03*	
C2	0.27617 (14)	1.18010 (13)	0.45379 (8)	0.0197 (2)	
H2	0.3173	1.2698	0.4449	0.024*	
C1	0.14425 (13)	1.11748 (13)	0.41362 (8)	0.0199 (2)	
H1	0.094	1.1659	0.3807	0.024*	
C20	−0.06275 (13)	1.06759 (13)	0.21126 (8)	0.0188 (2)	
H20	−0.0174	1.1566	0.2026	0.023*	
C22	−0.27381 (14)	0.89684 (15)	0.23717 (9)	0.0255 (3)	
H22	−0.3707	0.8694	0.2467	0.031*	
C4	0.29264 (14)	0.97544 (14)	0.51658 (8)	0.0216 (3)	
H4	0.3402	0.9279	0.5528	0.026*	
C17	0.42054 (13)	0.81708 (12)	0.06445 (8)	0.0166 (2)	
H17A	0.5191	0.8397	0.0478	0.02*	
H17B	0.4037	0.7463	0.1095	0.02*	
C8	0.20528 (14)	1.19726 (12)	0.05982 (8)	0.0187 (2)	
H8A	0.2067	1.1243	0.0188	0.022*	
H8B	0.1073	1.1941	0.0679	0.022*	
C29	0.13687 (16)	0.64750 (15)	0.65832 (10)	0.0294 (3)	
H29	0.2034	0.7136	0.6276	0.035*	
C25	−0.08721 (15)	0.49053 (14)	0.65995 (11)	0.0298 (3)	
H25	−0.174	0.4484	0.6301	0.036*	
C10	0.29188 (18)	1.44049 (13)	0.07790 (10)	0.0281 (3)	
H10A	0.1979	1.4486	0.0869	0.034*	
H10B	0.3476	1.5203	0.0485	0.034*	
C30	0.00960 (16)	0.58659 (15)	0.61498 (10)	0.0297 (3)	
H30	−0.0109	0.6108	0.5546	0.036*	
C11	0.35888 (16)	1.42569 (12)	0.17166 (10)	0.0257 (3)	
H11A	0.3598	1.4998	0.2123	0.031*	
H11B	0.4562	1.4266	0.1632	0.031*	
C27	0.06923 (17)	0.51601 (14)	0.79164 (10)	0.0281 (3)	
H27	0.0895	0.4918	0.8521	0.034*	
C28	0.16672 (16)	0.61169 (14)	0.74646 (10)	0.0288 (3)	
H28	0.2541	0.6528	0.7759	0.035*	
C26	−0.05788 (16)	0.45575 (13)	0.74830 (11)	0.0284 (3)	
H26	−0.125	0.3905	0.7793	0.034*	
C18	0.39160 (12)	0.93493 (11)	0.10898 (8)	0.0143 (2)	
H18A	0.4145	1.008	0.0658	0.017*	
H18B	0.4504	0.9612	0.1658	0.017*	
C14	0.14528 (13)	0.86015 (12)	0.04474 (8)	0.0176 (2)	
H14A	0.0465	0.838	0.0607	0.021*	
H14B	0.1641	0.9325	0.0009	0.021*	
C16	0.32791 (13)	0.77092 (12)	−0.02223 (8)	0.0180 (2)	
H16A	0.3521	0.8381	−0.0699	0.022*	
H16B	0.3447	0.6918	−0.0472	0.022*	
C31	0.52440 (16)	0.43687 (14)	0.57897 (10)	0.0283 (3)	
H31	0.541	0.3936	0.633	0.034*	
C32	0.59078 (16)	0.42566 (14)	0.49868 (11)	0.0293 (3)	
H32	0.6532	0.3748	0.4977	0.035*	
C33	0.43334 (16)	0.51160 (14)	0.58070 (11)	0.0291 (3)	
H33	0.3878	0.5197	0.6358	0.035*	

### Atomic displacement parameters (Å^2^)

**Table:**

	*U*^11^	*U*^22^	*U*^33^	*U*^12^	*U*^13^	*U*^23^
C13	0.0132 (5)	0.0144 (5)	0.0112 (5)	0.0050 (4)	−0.0004 (4)	−0.0011 (4)
Ru1	0.01057 (5)	0.01581 (5)	0.00970 (5)	0.00475 (3)	−0.00032 (3)	0.00017 (3)
Cl2	0.01653 (13)	0.01590 (12)	0.01587 (12)	0.00649 (10)	−0.00139 (10)	0.00159 (10)
Cl1	0.01180 (12)	0.01761 (13)	0.01576 (12)	0.00284 (10)	−0.00064 (10)	0.00031 (10)
P1	0.01084 (13)	0.01358 (13)	0.01068 (13)	0.00509 (10)	−0.00095 (10)	−0.00060 (10)
C21	0.0192 (6)	0.0392 (8)	0.0191 (6)	0.0170 (6)	−0.0003 (5)	−0.0004 (5)
C24	0.0154 (6)	0.0228 (6)	0.0218 (6)	0.0060 (5)	0.0005 (5)	−0.0028 (5)
C7	0.0158 (5)	0.0138 (5)	0.0134 (5)	0.0055 (4)	−0.0009 (4)	0.0000 (4)
C3	0.0196 (6)	0.0323 (7)	0.0105 (5)	0.0097 (5)	−0.0018 (4)	−0.0046 (5)
C19	0.0121 (5)	0.0222 (6)	0.0121 (5)	0.0067 (4)	−0.0013 (4)	−0.0019 (4)
C12	0.0259 (6)	0.0163 (5)	0.0156 (5)	0.0085 (5)	−0.0024 (5)	−0.0023 (4)
C15	0.0220 (6)	0.0181 (6)	0.0152 (5)	0.0064 (5)	−0.0021 (5)	−0.0037 (4)
C6	0.0143 (6)	0.0338 (7)	0.0138 (5)	0.0065 (5)	0.0035 (4)	−0.0019 (5)
C5	0.0214 (6)	0.0282 (7)	0.0150 (6)	0.0057 (5)	0.0069 (5)	0.0040 (5)
C9	0.0350 (7)	0.0187 (6)	0.0165 (6)	0.0108 (5)	0.0019 (5)	0.0030 (5)
C23	0.0160 (6)	0.0296 (7)	0.0247 (7)	0.0008 (5)	0.0011 (5)	−0.0029 (5)
C2	0.0232 (6)	0.0252 (6)	0.0128 (5)	0.0103 (5)	0.0004 (5)	−0.0049 (5)
C1	0.0191 (6)	0.0322 (7)	0.0128 (5)	0.0144 (5)	0.0012 (4)	−0.0027 (5)
C20	0.0181 (6)	0.0260 (6)	0.0150 (5)	0.0107 (5)	0.0000 (4)	−0.0001 (5)
C22	0.0122 (6)	0.0445 (8)	0.0195 (6)	0.0080 (6)	0.0002 (5)	−0.0022 (6)
C4	0.0234 (6)	0.0347 (7)	0.0100 (5)	0.0133 (6)	0.0024 (5)	0.0042 (5)
C17	0.0179 (6)	0.0187 (5)	0.0155 (5)	0.0086 (5)	0.0023 (4)	−0.0001 (4)
C8	0.0247 (6)	0.0181 (6)	0.0138 (5)	0.0075 (5)	−0.0029 (5)	0.0006 (4)
C29	0.0274 (7)	0.0277 (7)	0.0265 (7)	−0.0026 (6)	0.0052 (6)	0.0001 (6)
C25	0.0208 (7)	0.0254 (7)	0.0402 (8)	0.0026 (5)	−0.0036 (6)	−0.0017 (6)
C10	0.0477 (9)	0.0177 (6)	0.0219 (6)	0.0143 (6)	0.0010 (6)	0.0028 (5)
C30	0.0328 (8)	0.0284 (7)	0.0251 (7)	0.0048 (6)	−0.0028 (6)	0.0004 (6)
C11	0.0376 (8)	0.0147 (6)	0.0238 (7)	0.0066 (5)	−0.0025 (6)	−0.0016 (5)
C27	0.0383 (8)	0.0232 (6)	0.0234 (7)	0.0100 (6)	0.0012 (6)	−0.0002 (5)
C28	0.0249 (7)	0.0286 (7)	0.0280 (7)	0.0011 (6)	−0.0031 (6)	−0.0057 (6)
C26	0.0276 (7)	0.0185 (6)	0.0376 (8)	0.0036 (5)	0.0098 (6)	0.0035 (5)
C18	0.0143 (5)	0.0154 (5)	0.0138 (5)	0.0050 (4)	0.0018 (4)	−0.0001 (4)
C14	0.0184 (6)	0.0212 (6)	0.0148 (5)	0.0089 (5)	−0.0043 (4)	−0.0049 (4)
C16	0.0237 (6)	0.0182 (6)	0.0141 (5)	0.0094 (5)	0.0016 (5)	−0.0017 (4)
C31	0.0353 (8)	0.0228 (6)	0.0273 (7)	0.0108 (6)	−0.0125 (6)	−0.0045 (5)
C32	0.0307 (7)	0.0253 (7)	0.0353 (8)	0.0147 (6)	−0.0106 (6)	−0.0079 (6)
C33	0.0328 (8)	0.0269 (7)	0.0283 (7)	0.0106 (6)	−0.0043 (6)	−0.0071 (6)

### Geometric parameters (Å, º)

**Table:**

C13—C18	1.5352 (16)	C23—C22	1.393 (2)
C13—C14	1.5422 (15)	C23—H23	0.95
C13—P1	1.8536 (11)	C2—C1	1.4111 (18)
C13—H13	1	C2—H2	0.95
Ru1—C5	2.1708 (13)	C1—H1	0.95
Ru1—C1	2.1808 (12)	C20—H20	0.95
Ru1—C6	2.1816 (13)	C22—H22	0.95
Ru1—C2	2.1919 (12)	C4—H4	0.95
Ru1—C4	2.2667 (12)	C17—C16	1.5328 (17)
Ru1—C3	2.2677 (12)	C17—C18	1.5333 (16)
Ru1—P1	2.3786 (3)	C17—H17A	0.99
Ru1—Cl1	2.4137 (3)	C17—H17B	0.99
Ru1—Cl2	2.4239 (3)	C8—H8A	0.99
P1—C19	1.8312 (12)	C8—H8B	0.99
P1—C7	1.8564 (12)	C29—C28	1.387 (2)
C21—C22	1.387 (2)	C29—C30	1.389 (2)
C21—C20	1.3993 (18)	C29—H29	0.95
C21—H21	0.95	C25—C30	1.384 (2)
C24—C23	1.3925 (18)	C25—C26	1.385 (2)
C24—C19	1.4067 (18)	C25—H25	0.95
C24—H24	0.95	C10—C11	1.5295 (19)
C7—C12	1.5422 (16)	C10—H10A	0.99
C7—C8	1.5429 (16)	C10—H10B	0.99
C7—H7	1	C30—H30	0.95
C3—C4	1.388 (2)	C11—H11A	0.99
C3—C2	1.4351 (18)	C11—H11B	0.99
C3—H3	0.95	C27—C26	1.387 (2)
C19—C20	1.3986 (17)	C27—C28	1.387 (2)
C12—C11	1.5318 (18)	C27—H27	0.95
C12—H12A	0.99	C28—H28	0.95
C12—H12B	0.99	C26—H26	0.95
C15—C16	1.5290 (18)	C18—H18A	0.99
C15—C14	1.5366 (16)	C18—H18B	0.99
C15—H15A	0.99	C14—H14A	0.99
C15—H15B	0.99	C14—H14B	0.99
C6—C5	1.4074 (19)	C16—H16A	0.99
C6—C1	1.426 (2)	C16—H16B	0.99
C6—H6	0.95	C31—C32	1.382 (2)
C5—C4	1.4357 (19)	C31—C33	1.391 (2)
C5—H5	0.95	C31—H31	0.95
C9—C10	1.5249 (18)	C32—C33^i^	1.391 (2)
C9—C8	1.5330 (18)	C32—H32	0.95
C9—H9A	0.99	C33—C32^i^	1.391 (2)
C9—H9B	0.99	C33—H33	0.95
			
C18—C13—C14	110.24 (9)	H9A—C9—H9B	107.9
C18—C13—P1	111.93 (8)	C24—C23—C22	120.14 (13)
C14—C13—P1	118.23 (8)	C24—C23—H23	119.9
C18—C13—H13	105.1	C22—C23—H23	119.9
C14—C13—H13	105.1	C1—C2—C3	119.86 (12)
P1—C13—H13	105.1	C1—C2—Ru1	70.74 (7)
C5—Ru1—C1	68.40 (5)	C3—C2—Ru1	74.12 (7)
C5—Ru1—C6	37.73 (5)	C1—C2—H2	120.1
C1—Ru1—C6	38.15 (5)	C3—C2—H2	120.1
C5—Ru1—C2	80.54 (5)	Ru1—C2—H2	127
C1—Ru1—C2	37.65 (5)	C2—C1—C6	119.93 (12)
C6—Ru1—C2	68.32 (5)	C2—C1—Ru1	71.60 (7)
C5—Ru1—C4	37.68 (5)	C6—C1—Ru1	70.96 (7)
C1—Ru1—C4	79.39 (5)	C2—C1—H1	120
C6—Ru1—C4	67.44 (5)	C6—C1—H1	120
C2—Ru1—C4	66.63 (5)	Ru1—C1—H1	129.9
C5—Ru1—C3	66.62 (5)	C19—C20—C21	120.63 (13)
C1—Ru1—C3	67.21 (5)	C19—C20—H20	119.7
C6—Ru1—C3	79.24 (5)	C21—C20—H20	119.7
C2—Ru1—C3	37.50 (5)	C21—C22—C23	119.51 (12)
C4—Ru1—C3	35.64 (5)	C21—C22—H22	120.2
C5—Ru1—P1	121.28 (4)	C23—C22—H22	120.2
C1—Ru1—P1	90.39 (3)	C3—C4—C5	119.47 (12)
C6—Ru1—P1	93.12 (3)	C3—C4—Ru1	72.22 (7)
C2—Ru1—P1	115.10 (3)	C5—C4—Ru1	67.54 (7)
C4—Ru1—P1	158.87 (4)	C3—C4—H4	120.3
C3—Ru1—P1	152.44 (4)	C5—C4—H4	120.3
C5—Ru1—Cl1	151.18 (4)	Ru1—C4—H4	133
C1—Ru1—Cl1	120.39 (4)	C16—C17—C18	111.25 (10)
C6—Ru1—Cl1	158.52 (4)	C16—C17—H17A	109.4
C2—Ru1—Cl1	92.13 (4)	C18—C17—H17A	109.4
C4—Ru1—Cl1	114.00 (4)	C16—C17—H17B	109.4
C3—Ru1—Cl1	90.67 (4)	C18—C17—H17B	109.4
P1—Ru1—Cl1	87.124 (11)	H17A—C17—H17B	108
C5—Ru1—Cl2	86.78 (4)	C9—C8—C7	111.20 (10)
C1—Ru1—Cl2	151.53 (4)	C9—C8—H8A	109.4
C6—Ru1—Cl2	113.39 (4)	C7—C8—H8A	109.4
C2—Ru1—Cl2	153.91 (3)	C9—C8—H8B	109.4
C4—Ru1—Cl2	89.50 (4)	C7—C8—H8B	109.4
C3—Ru1—Cl2	116.42 (3)	H8A—C8—H8B	108
P1—Ru1—Cl2	90.969 (10)	C28—C29—C30	119.92 (14)
Cl1—Ru1—Cl2	88.071 (11)	C28—C29—H29	120
C19—P1—C13	103.21 (5)	C30—C29—H29	120
C19—P1—C7	110.18 (5)	C30—C25—C26	120.13 (14)
C13—P1—C7	106.93 (5)	C30—C25—H25	119.9
C19—P1—Ru1	109.13 (4)	C26—C25—H25	119.9
C13—P1—Ru1	113.89 (4)	C9—C10—C11	111.01 (11)
C7—P1—Ru1	113.03 (4)	C9—C10—H10A	109.4
C22—C21—C20	120.56 (12)	C11—C10—H10A	109.4
C22—C21—H21	119.7	C9—C10—H10B	109.4
C20—C21—H21	119.7	C11—C10—H10B	109.4
C23—C24—C19	121.02 (12)	H10A—C10—H10B	108
C23—C24—H24	119.5	C25—C30—C29	119.91 (14)
C19—C24—H24	119.5	C25—C30—H30	120
C12—C7—C8	110.59 (9)	C29—C30—H30	120
C12—C7—P1	114.10 (8)	C10—C11—C12	111.49 (12)
C8—C7—P1	115.61 (8)	C10—C11—H11A	109.3
C12—C7—H7	105.1	C12—C11—H11A	109.3
C8—C7—H7	105.1	C10—C11—H11B	109.3
P1—C7—H7	105.1	C12—C11—H11B	109.3
C4—C3—C2	120.40 (12)	H11A—C11—H11B	108
C4—C3—Ru1	72.14 (7)	C26—C27—C28	119.83 (14)
C2—C3—Ru1	68.39 (7)	C26—C27—H27	120.1
C4—C3—H3	119.8	C28—C27—H27	120.1
C2—C3—H3	119.8	C27—C28—C29	120.08 (14)
Ru1—C3—H3	132.8	C27—C28—H28	120
C20—C19—C24	118.14 (11)	C29—C28—H28	120
C20—C19—P1	124.07 (10)	C25—C26—C27	120.12 (14)
C24—C19—P1	117.23 (9)	C25—C26—H26	119.9
C11—C12—C7	110.46 (10)	C27—C26—H26	119.9
C11—C12—H12A	109.6	C17—C18—C13	109.57 (10)
C7—C12—H12A	109.6	C17—C18—H18A	109.8
C11—C12—H12B	109.6	C13—C18—H18A	109.8
C7—C12—H12B	109.6	C17—C18—H18B	109.8
H12A—C12—H12B	108.1	C13—C18—H18B	109.8
C16—C15—C14	111.35 (10)	H18A—C18—H18B	108.2
C16—C15—H15A	109.4	C15—C14—C13	109.76 (10)
C14—C15—H15A	109.4	C15—C14—H14A	109.7
C16—C15—H15B	109.4	C13—C14—H14A	109.7
C14—C15—H15B	109.4	C15—C14—H14B	109.7
H15A—C15—H15B	108	C13—C14—H14B	109.7
C5—C6—C1	119.38 (12)	H14A—C14—H14B	108.2
C5—C6—Ru1	70.72 (7)	C15—C16—C17	111.11 (10)
C1—C6—Ru1	70.89 (7)	C15—C16—H16A	109.4
C5—C6—H6	120.3	C17—C16—H16A	109.4
C1—C6—H6	120.3	C15—C16—H16B	109.4
Ru1—C6—H6	130.6	C17—C16—H16B	109.4
C6—C5—C4	120.66 (13)	H16A—C16—H16B	108
C6—C5—Ru1	71.55 (7)	C32—C31—C33	119.98 (14)
C4—C5—Ru1	74.79 (7)	C32—C31—H31	120
C6—C5—H5	119.7	C33—C31—H31	120
C4—C5—H5	119.7	C31—C32—C33^i^	120.33 (14)
Ru1—C5—H5	125.8	C31—C32—H32	119.8
C10—C9—C8	111.74 (11)	C33^i^—C32—H32	119.8
C10—C9—H9A	109.3	C32^i^—C33—C31	119.69 (15)
C8—C9—H9A	109.3	C32^i^—C33—H33	120.2
C10—C9—H9B	109.3	C31—C33—H33	120.2
C8—C9—H9B	109.3		
			
C18—C13—P1—C19	169.93 (8)	P1—Ru1—C5—C4	−177.46 (6)
C14—C13—P1—C19	40.18 (10)	Cl1—Ru1—C5—C4	13.27 (13)
C18—C13—P1—C7	53.70 (9)	Cl2—Ru1—C5—C4	93.36 (7)
C14—C13—P1—C7	−76.05 (10)	C19—C24—C23—C22	−0.8 (2)
C18—C13—P1—Ru1	−71.90 (8)	C4—C3—C2—C1	3.99 (18)
C14—C13—P1—Ru1	158.35 (8)	Ru1—C3—C2—C1	56.04 (10)
C5—Ru1—P1—C19	21.52 (6)	C4—C3—C2—Ru1	−52.05 (11)
C1—Ru1—P1—C19	−43.22 (6)	C5—Ru1—C2—C1	−66.72 (8)
C6—Ru1—P1—C19	−5.15 (6)	C6—Ru1—C2—C1	−29.53 (8)
C2—Ru1—P1—C19	−72.63 (6)	C4—Ru1—C2—C1	−103.39 (9)
C4—Ru1—P1—C19	17.21 (11)	C3—Ru1—C2—C1	−130.37 (12)
C3—Ru1—P1—C19	−77.71 (9)	P1—Ru1—C2—C1	53.49 (8)
Cl1—Ru1—P1—C19	−163.64 (4)	Cl1—Ru1—C2—C1	141.32 (7)
Cl2—Ru1—P1—C19	108.34 (4)	Cl2—Ru1—C2—C1	−128.72 (8)
C5—Ru1—P1—C13	−93.20 (6)	C5—Ru1—C2—C3	63.64 (8)
C1—Ru1—P1—C13	−157.94 (6)	C1—Ru1—C2—C3	130.37 (12)
C6—Ru1—P1—C13	−119.87 (6)	C6—Ru1—C2—C3	100.84 (9)
C2—Ru1—P1—C13	172.65 (6)	C4—Ru1—C2—C3	26.98 (8)
C4—Ru1—P1—C13	−97.51 (11)	P1—Ru1—C2—C3	−176.14 (7)
C3—Ru1—P1—C13	167.57 (8)	Cl1—Ru1—C2—C3	−88.32 (8)
Cl1—Ru1—P1—C13	81.64 (4)	Cl2—Ru1—C2—C3	1.64 (14)
Cl2—Ru1—P1—C13	−6.38 (4)	C3—C2—C1—C6	−3.71 (18)
C5—Ru1—P1—C7	144.49 (6)	Ru1—C2—C1—C6	53.97 (10)
C1—Ru1—P1—C7	79.75 (6)	C3—C2—C1—Ru1	−57.67 (10)
C6—Ru1—P1—C7	117.83 (6)	C5—C6—C1—C2	−0.86 (18)
C2—Ru1—P1—C7	50.34 (6)	Ru1—C6—C1—C2	−54.27 (10)
C4—Ru1—P1—C7	140.19 (11)	C5—C6—C1—Ru1	53.41 (10)
C3—Ru1—P1—C7	45.26 (9)	C5—Ru1—C1—C2	102.95 (9)
Cl1—Ru1—P1—C7	−40.66 (4)	C6—Ru1—C1—C2	132.15 (11)
Cl2—Ru1—P1—C7	−128.68 (4)	C4—Ru1—C1—C2	65.31 (8)
C19—P1—C7—C12	80.39 (9)	C3—Ru1—C1—C2	30.20 (8)
C13—P1—C7—C12	−168.11 (8)	P1—Ru1—C1—C2	−133.29 (7)
Ru1—P1—C7—C12	−41.99 (9)	Cl1—Ru1—C1—C2	−46.40 (8)
C19—P1—C7—C8	−49.53 (10)	Cl2—Ru1—C1—C2	133.96 (7)
C13—P1—C7—C8	61.97 (10)	C5—Ru1—C1—C6	−29.20 (7)
Ru1—P1—C7—C8	−171.91 (7)	C2—Ru1—C1—C6	−132.15 (11)
C5—Ru1—C3—C4	28.75 (8)	C4—Ru1—C1—C6	−66.84 (8)
C1—Ru1—C3—C4	104.07 (9)	C3—Ru1—C1—C6	−101.94 (8)
C6—Ru1—C3—C4	66.10 (8)	P1—Ru1—C1—C6	94.57 (7)
C2—Ru1—C3—C4	134.39 (12)	Cl1—Ru1—C1—C6	−178.54 (6)
P1—Ru1—C3—C4	141.96 (7)	Cl2—Ru1—C1—C6	1.81 (12)
Cl1—Ru1—C3—C4	−132.98 (7)	C24—C19—C20—C21	0.37 (18)
Cl2—Ru1—C3—C4	−44.80 (8)	P1—C19—C20—C21	−170.69 (10)
C5—Ru1—C3—C2	−105.64 (9)	C22—C21—C20—C19	−0.27 (19)
C1—Ru1—C3—C2	−30.32 (8)	C20—C21—C22—C23	−0.4 (2)
C6—Ru1—C3—C2	−68.29 (8)	C24—C23—C22—C21	0.9 (2)
C4—Ru1—C3—C2	−134.39 (12)	C2—C3—C4—C5	0.32 (18)
P1—Ru1—C3—C2	7.57 (13)	Ru1—C3—C4—C5	−50.06 (10)
Cl1—Ru1—C3—C2	92.63 (8)	C2—C3—C4—Ru1	50.38 (11)
Cl2—Ru1—C3—C2	−179.19 (7)	C6—C5—C4—C3	−4.96 (18)
C23—C24—C19—C20	0.15 (18)	Ru1—C5—C4—C3	52.19 (11)
C23—C24—C19—P1	171.83 (10)	C6—C5—C4—Ru1	−57.14 (10)
C13—P1—C19—C20	−142.05 (10)	C5—Ru1—C4—C3	−133.75 (12)
C7—P1—C19—C20	−28.16 (12)	C1—Ru1—C4—C3	−65.49 (8)
Ru1—P1—C19—C20	96.50 (10)	C6—Ru1—C4—C3	−103.44 (9)
C13—P1—C19—C24	46.81 (11)	C2—Ru1—C4—C3	−28.28 (8)
C7—P1—C19—C24	160.70 (9)	P1—Ru1—C4—C3	−127.73 (10)
Ru1—P1—C19—C24	−74.64 (10)	Cl1—Ru1—C4—C3	53.20 (8)
C8—C7—C12—C11	−56.30 (14)	Cl2—Ru1—C4—C3	140.87 (7)
P1—C7—C12—C11	171.32 (9)	C1—Ru1—C4—C5	68.27 (8)
C1—Ru1—C6—C5	−132.16 (11)	C6—Ru1—C4—C5	30.32 (8)
C2—Ru1—C6—C5	−102.99 (9)	C2—Ru1—C4—C5	105.47 (9)
C4—Ru1—C6—C5	−30.27 (8)	C3—Ru1—C4—C5	133.75 (12)
C3—Ru1—C6—C5	−65.51 (8)	P1—Ru1—C4—C5	6.03 (15)
P1—Ru1—C6—C5	141.19 (7)	Cl1—Ru1—C4—C5	−173.04 (7)
Cl1—Ru1—C6—C5	−128.73 (9)	Cl2—Ru1—C4—C5	−85.38 (8)
Cl2—Ru1—C6—C5	48.78 (8)	C10—C9—C8—C7	−55.01 (15)
C5—Ru1—C6—C1	132.16 (11)	C12—C7—C8—C9	55.45 (14)
C2—Ru1—C6—C1	29.17 (7)	P1—C7—C8—C9	−172.95 (9)
C4—Ru1—C6—C1	101.89 (8)	C8—C9—C10—C11	55.02 (17)
C3—Ru1—C6—C1	66.65 (7)	C26—C25—C30—C29	0.5 (2)
P1—Ru1—C6—C1	−86.65 (7)	C28—C29—C30—C25	0.2 (2)
Cl1—Ru1—C6—C1	3.44 (14)	C9—C10—C11—C12	−56.16 (17)
Cl2—Ru1—C6—C1	−179.06 (6)	C7—C12—C11—C10	56.94 (15)
C1—C6—C5—C4	5.21 (18)	C26—C27—C28—C29	0.3 (2)
Ru1—C6—C5—C4	58.71 (11)	C30—C29—C28—C27	−0.6 (2)
C1—C6—C5—Ru1	−53.49 (10)	C30—C25—C26—C27	−0.7 (2)
C1—Ru1—C5—C6	29.50 (8)	C28—C27—C26—C25	0.3 (2)
C2—Ru1—C5—C6	66.63 (8)	C16—C17—C18—C13	57.64 (13)
C4—Ru1—C5—C6	130.38 (12)	C14—C13—C18—C17	−59.41 (12)
C3—Ru1—C5—C6	103.09 (9)	P1—C13—C18—C17	166.81 (8)
P1—Ru1—C5—C6	−47.08 (9)	C16—C15—C14—C13	−56.64 (13)
Cl1—Ru1—C5—C6	143.65 (7)	C18—C13—C14—C15	58.94 (13)
Cl2—Ru1—C5—C6	−136.26 (8)	P1—C13—C14—C15	−170.54 (8)
C1—Ru1—C5—C4	−100.88 (9)	C14—C15—C16—C17	55.08 (13)
C6—Ru1—C5—C4	−130.38 (12)	C18—C17—C16—C15	−55.60 (13)
C2—Ru1—C5—C4	−63.75 (8)	C33—C31—C32—C33^i^	−0.2 (2)
C3—Ru1—C5—C4	−27.30 (8)	C32—C31—C33—C32^i^	0.2 (2)

Symmetry code: (i) −*x*+1, −*y*+1, −*z*+1.

### Hydrogen-bond geometry (Å, º)

Cg1 and Cg2 are the centroids of the C19–C24 and C31–C33/C31'–C33') benzene rings.

**Table:**

*D*—H···*A*	*D*—H	H···*A*	*D*···*A*	*D*—H···*A*
C3—H3···Cl2^ii^	0.95	2.76	3.6307 (13)	153
C4—H4···Cl1^ii^	0.95	2.7	3.6209 (13)	163
C6—H6···*Cg*1	0.95	2.78	3.5086 (14)	135
C2—H2···*Cg*2^iii^	0.95	2.73	3.5869 (15)	150

Symmetry codes: (ii) −*x*+1, −*y*+2, −*z*+1; (iii) *x*, *y*+1, *z*.
